# Balloon atrial septostomy: a weapon to challenge right heart failure after cardiac surgery

**DOI:** 10.1186/s13019-024-02884-8

**Published:** 2024-07-01

**Authors:** Weijun Yang, Zhean Shen, Manxuan Zhu, Xiaofang Wang, Minjian Kong

**Affiliations:** 1 Department of Cardiovascular Surgery, The Second Affiliated Hospital of Zhejiang University School of Medicine, Hangzhou, Zhejiang 310009 P.R. China; 2 School of Medicine, Zhejiang University, Hangzhou, Zhejiang 310000 P.R. China; 3 The First School of Clinical Medicine, Zhejiang Chinese Medical University, Hangzhou, Zhejiang 310053 P.R. China

**Keywords:** Heart failure, Cardiac surgery, Balloon atrial septostomy, Case report

## Abstract

Right heart failure is a common complication after cardiac surgery, and its mortality remains high. The medical management and veno-arterial extracorporeal membrane oxygenation has shown significant improvement in the majority of cases. However, a minority of patients may still require long-term mechanical circulatory support or heart transplantation. Balloon atrial septostomy is a new method for the prevention and treatment of right heart failure, which may avoid the patient’s dependence on mechanical circulatory support. We used this method to try to treat patients with right heart failure after cardiac surgery, and all received good benefits. Therefore, we selected several representative cases to report, in order to guide other qualified cardiac surgeons to carry out relevant clinical practice.

**Supplementary Information:**

The online version contains supplementary material available at 10.1186/s13019-024-02884-8.

## Introduction

Approximately 3% of patients may experience post-operative right ventricular failure following cardiac surgery [1]. The variables that exhibite a significant association with right ventricular failure include right coronary occlusion, valvular heart disease (particularly in the presence of pulmonary hypertension), heart transplantation, LVAD insertion, and non-homogeneous distribution of cardioplegia [2, 3]. The prevalence of severe right ventricular failure following cardiotomy is estimated to be 0.1% [4]. The medical management of right ventricular failure encompasses various strategies, such as the administration of inotropic agents to enhance cardiac contractility, optimization of right ventricular preload, reduction of right ventricular afterload through the use of inhaled nitric oxide, and the maintenance of sinus rhythm Veno-Arterial Extracorporeal Membrane Oxygenation (VA-ECMO) can be considered as an effective intervention for patients who are facing acute right ventricular failure that does not respond to conventional medical therapy. The right ventricular function exhibited gradual improvement in the majority of patients, leading to the successful removal of VA-ECMO. However, a minority of patients may encounter challenges in the process of weaning from VA-ECMO, necessitating long-term mechanical circulatory support or heart transplantation [5–7].

Atrial septostomy has been employed in clinical settings since the **1960s** as a palliative therapy for cyanotic heart disease and refractory primary pulmonary hypertension [8–10]. In this research, atrial septostomy was utilized to improve the management of weaning from VA-ECMO in patients with post-cardiotomy right ventricular failure. The present report aims to share our experience with a group of four patients. The report has been granted approval by the Ethics Committee of the Second Affiliated Hospital of Zhejiang University School of Medicine.

### Case presentations

#### Case 1

**Fig. 1:**
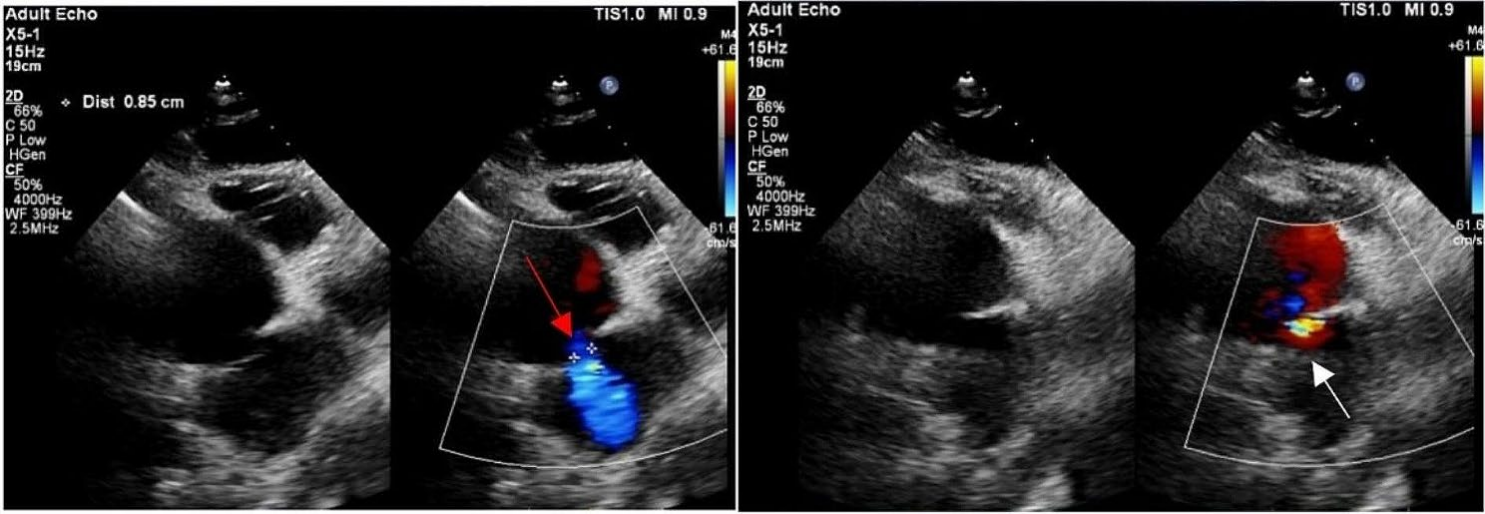
Echocardiography showing atrial septal defect and blood flow after Balloon atrial septostomy. The red arrow indicates a right to left shunt of blood flow, and the white arrow indicates a left to right shunt of blood flow

A 71 years old male patient presented with chest tightness for a month. The patient underwent a cardiac catheterization at a local hospital, which confirmed the presence of an 80% thrombotic lesion in the right coronary artery. The right coronary artery perforation ocuured and he developed cardaic tamponade. He was transferred urgently to our facility for emergence surgical intervention. The right coronary artery was anastomosed to a segment of the saphenous vein, and the ruptured coronary arteries were subsequently repaired. After achieving sufficient reperfusion, it was observed that the contractility of the right ventricle continued to be significantly compromised. The patient was promptly commenced on VA-ECMO. Following 8 days of VA-ECMO, the patient exhibited stable hemodynamics with low doses of inotropic for a duration of 24 h. Additionally, the patient maintained a mean arterial pressure exceeding 60 mmHg, displayed a left ventricular ejection fraction of 30%, and exhibited a tricuspid annular plane systolic excursion (TAPSE) of 1.2 cm. The central venous pressure (CVP) exhibited an increase from 12mmHg to 18mmHg upon reducing VA-ECMO flow to 1.2 L. Following the weaning of VA-ECMO, CVP exhibited a subsequent elevation to 25mmHg, accompanied by recurrent episodes of rapid atrial fibrillation, hypotension, and oliguria. These manifestations ultimately led to second attack of cardiogenic shock, necessitating the resumption of VA-ECMO support after a lapse of 14 h. Subsequent to the aforementioned occurrences, the patient relied VA-ECMO.Then, he underwent a Balloon atrial septostomy on the fourteenth day post-cardiac surgery. The patient’s CVP was decreased to 15mmHg, while the peripheral capillary oxygen saturation (SpO2) at 93%. Echocardiography revealed a left ventricular ejection fraction of 45%, TAPSE of 0.7 cm, and a right ventricular ejection fraction (RVEF) of 28%. Additionally, right ventricular systolic dysfunction with severe hypokinesis in the middle and upper areas was observed, along with the presence of a 1.03 cm inter-atrial shunting from the right to the left side. VA-ECMO was weaned after a single day. The patient was transferred out from the intensive care unit after 40 days, discharged one week after that. One year later, a follow-up echocardiography revealed TAPSE of 0.95 cm, indicating reduced movement of the right ventricular free wall. There was a bidirectional diversion of 0.85 cm at the atrial septal defect. During the recent telephone follow-up, the patient’s heart function was categorized as Class II according to the New York Heart Association(NYHA)Classification (Fig. 1).

## Case 2

**Fig. 2:**
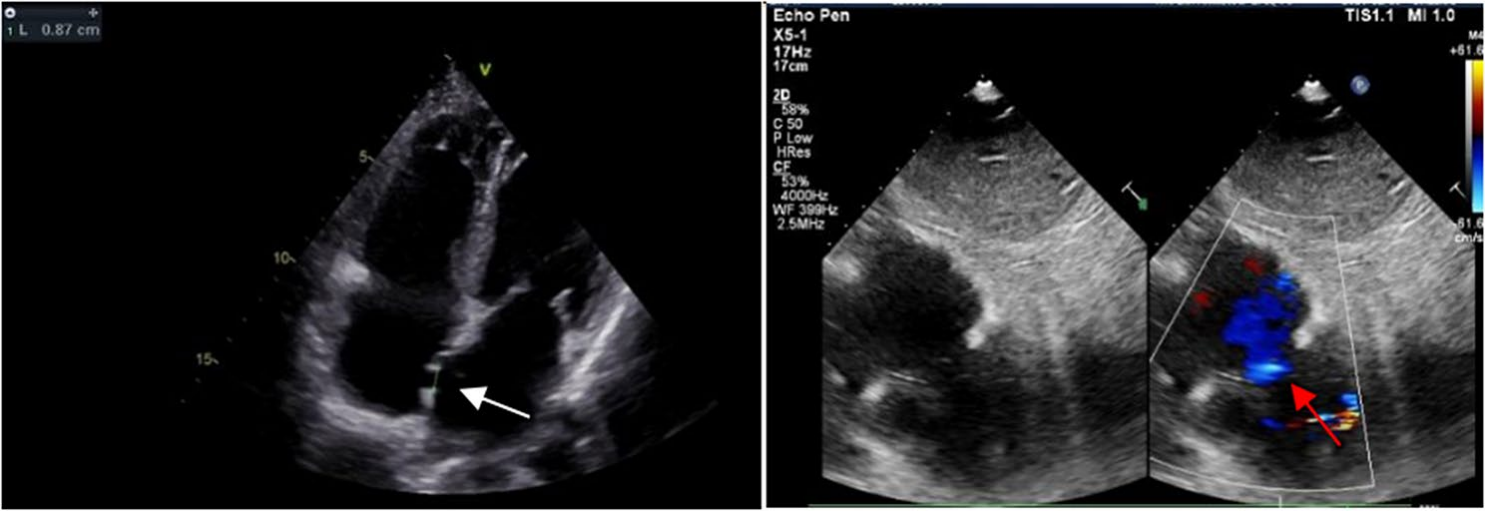
Echocardiography showing atrial septal defect and blood flow after Balloon atrial septostomy. The white arrow indicates atrial septal defect, and the red arrow indicates a right to left shunt of blood flow

A male patient, 51 years old, presented with shortness of breath for 2 years, which had exacerbated over the course of the past 10 days. The patient’s New York Heart Association (NYHA) classification was class 3. Transthoracic echocardiography revealed a tendon rupture of the mitral valve, anterior mitral valve prolapse in zone A3, and severe regurgitation. Left ventricular EF of 65%. The right atrium and right ventricle displayed normal size and function, with minimal regurgitation of the tricuspid valve. The thoracoscopic mitral valve repair procedure was performed, utilizing del Nido solution as cardioplegia. The durations of cardiopulmonary bypass and aortic cross-clamping were recorded as 300 and 239 min, respectively. The patient made two unsuccessful attempts to withdraw from cardiopulmonary bypass machine before ultimately achieving success. Then he was send to the intensive care unit. Following the conclusion of the surgical procedure, the commencement of administering elevated dosages of cardiac stimulants was initiated, encompassing epinephrine at a rate of 0.2 µg/kg/min, dobutamine at a rate of 20 µg/kg/min, and norepinephrine at a level of 0.1 µg/kg/min. The Swan-Ganz catheter provided measurements indicating a mean pulmonary artery pressure (MPAP) of 25 mmHg, pulmonary artery wedge pressure (PAWP) of 12 mmHg, and a cardiac index of 2.12 L/min/m^2^. The bedside Transthoracic echocardiography demonstrated normal left ventricular systolic function, a notable decline in right ventricular systolic function, moderate regurgitation in both mitral and tricuspid valves. Moreover, there exists substantiating evidence of impaired hepatic and renal functionality. In response to the persistent instability of vital signs and the manifestation of right ventricular dysfunction, VA-ECMO was initiated on the initial day following the surgical procedure. Following the implementation of VA-ECMO support, the persistent metabolic acidosis was successfully corrected, resulting in the stabilization of vital signs. After a period of 6 days of the support, the patient has become dependent on VA-ECMO. Coronary angiography was conducted on the eighth day after VA-ECMO support to confirm the absence of lesions in all three coronary arteries. Additionally, balloon atrial septostomy was performed concurrently. The echocardiography follow-up revealed a significant reduction in activity in the right ventricular free wall, as indicated by TAPSE of 0.87 cm.Notably, there was a presence of regurgitation in the tricuspid valve. Furthermore, a bidirectional split blood stream measuring 0.87 cm was observed at the atrial septum, primarily flowing from the right to the left. Subsequently, the process of weaning from VA-ECMO was initiated on the following day. After a month, the patient was transferred from the intensive care unit, and he was discharged home 48 days postoperatively (Fig. 2).

## Case 3

The patient, a 60-year-old woman, presented with symptoms of dyspnea, abdominal distention, and lower extremity edema that had manifested two weeks prior to her consultation. She had a decade-long medical record of constrictive pericarditis. Transthoracic echocardiogram that revealed findings including a mildly thickened pericardium, small pericardial effusion, dilated inferior vena cava and hepatic veins, severe tricuspid regurgitation, TAPSE measuring 1.80 cm, RV FAC of 39.0%, and the inner diameter of the tricuspid annulus measured 4.3 cm. The patient underwent an initial treatment regimen involving the intravenous administration of frusemide and spironolactone, resulting in the resolution of edema in both lower limbs and alleviation of patient symptoms. The pericardiectomy and tricuspid plasty procedures were performed two weeks after the initial treatment, resulting in a reduction of the CVP from 25mmHg to 13mmHg. The patient’s recovery following the surgery was uncomplicated, leading to her transfer from the intensive care unit on the second day after the operation. After a span of two days, the patient was once again relocated to the ICU due to the presence of hypotension and oliguria. The patient exhibiting acute right ventricular failure demonstrated resistance to conventional medical interventions, necessitating the implementation of VA-ECMO. Transthoracic echocardiogram revealed minimal regurgitation of both the tricuspid valve and the mitral valve, abnormal segmental movement of the right ventricular myocardium, and right cardiac insufficiency (TAPSE, 0.83 cm; RV FAC, 23%). On the eighth day of VA-ECMO support, it was observed that the right cardiac function remained inadequate. Consequently, balloon atrial septostomy procedure was conducted. The VA-ECMO support was subsequently discontinued on the next day, and the patient was transferred out of the intensive care unit after two weeks. One month after discharge, a follow-up cardiac ultrasound was conducted, which indicated bidirectional septal blood flow measuring approximately 0.68 cm from left to right at the atrial septum. Furthermore, minor tricuspid regurgitation was observed, along with RV FAC of 28%.

## Case 4

A 59-year-old female patient was admitted to the hospital as a result of recurrent mitral stenosis. The patient had undergone mitral valve replacement surgery three decades ago, although the specific type and size of the prosthetic valve was unknown. Transesophageal echocardiography demonstrated a significant degree of mitral valve stenosis, mild tricuspid regurgitation, pulmonary hypertension with a Pulmonary artery systolic pressure (PASP) of 57mmHg, impaired right heart function, TAPSE of 1.33 cm, and RV FAC of 25%. The patient underwent a subsequent mitral valve replacement procedure, during which it was ascertained that the occurrence of mitral stenosis was attributed to pannus formation beneath the mechanical prosthesis within the mitral valve. The patient experienced a satisfactory postoperative recovery and was discharged after one week. However, after one month, the patient presented with symptoms indicative of pleural effusion, fatigue, oliguria, and impaired liver and renal function. The administration of diuretic, vasoactive, and inotropic agents yielded a positive systemic response, effectively decreasing preload on the right ventricle and subsequently ameliorating the patient’s condition. Transthoracic echocardiogram revealed a normal transmitral pressure gradient, severe tricuspid regurgitation, pulmonary hypertension (PASP 56mmHg), TAPSE 1.6 cm. However, three weeks subsequent to admission, the patient encountered a deterioration in hercondition, marked by hypotension arising from recurrent atrial tachycardia. Despite administering higher doses of vasoactive and inotropic agents, the patient’s condition did not improve. Additionally, the patient exhibited signs of liver and kidney insufficiency. When administered at the specified rates of 20 µg/kg/min for dobutamine, 1 µg/kg/min for milrinone, and 0.1 µg/kg/min for norepinephrine, Swan- Ganz monitoring data yielded the following values: CVP of 30mmHg, pulmonary artery pressure (PAP) of 60/34mmHg, cardiac output of 3.3 L/min, cardiac index of 2.1/(min·m)^**2**^, and PAWP) of 12mmHg. The patient underwent femoral arterial and venous cannulation for the implementation of VA-ECMO, alongside the administration of continuous renal replacement therapy. Following the attainment of a stable mean arterial pressure within the range of 50 to 70 mm Hg and the restoration of normal sinus rhythm with a heart rate of 70 to 110 bpm, the gradual discontinuation of vasoactive agents was initiated. Following a 6-day treatment period, an attempt was made to reduce the flow rate to 1.5 L/min. This adjustment led to a notable increase in CVP and a significant decrease in blood pressure. In response, balloon atrial septostomy was performed, and subsequent echocardiography revealed a substantial presence of tricuspid regurgitation. Regrettably, the patient exhibited lung infection, which subsequently led to septic shock on the 8th day of VA-ECMO assistance. In response to the deteriorating condition, patient’s family expressed their desire to discontinue VA-ECMO, and due to the inability to sustain blood pressure, the treatment was ultimately terminated.

### Balloon atrial septostomy

**Fig. 3:**
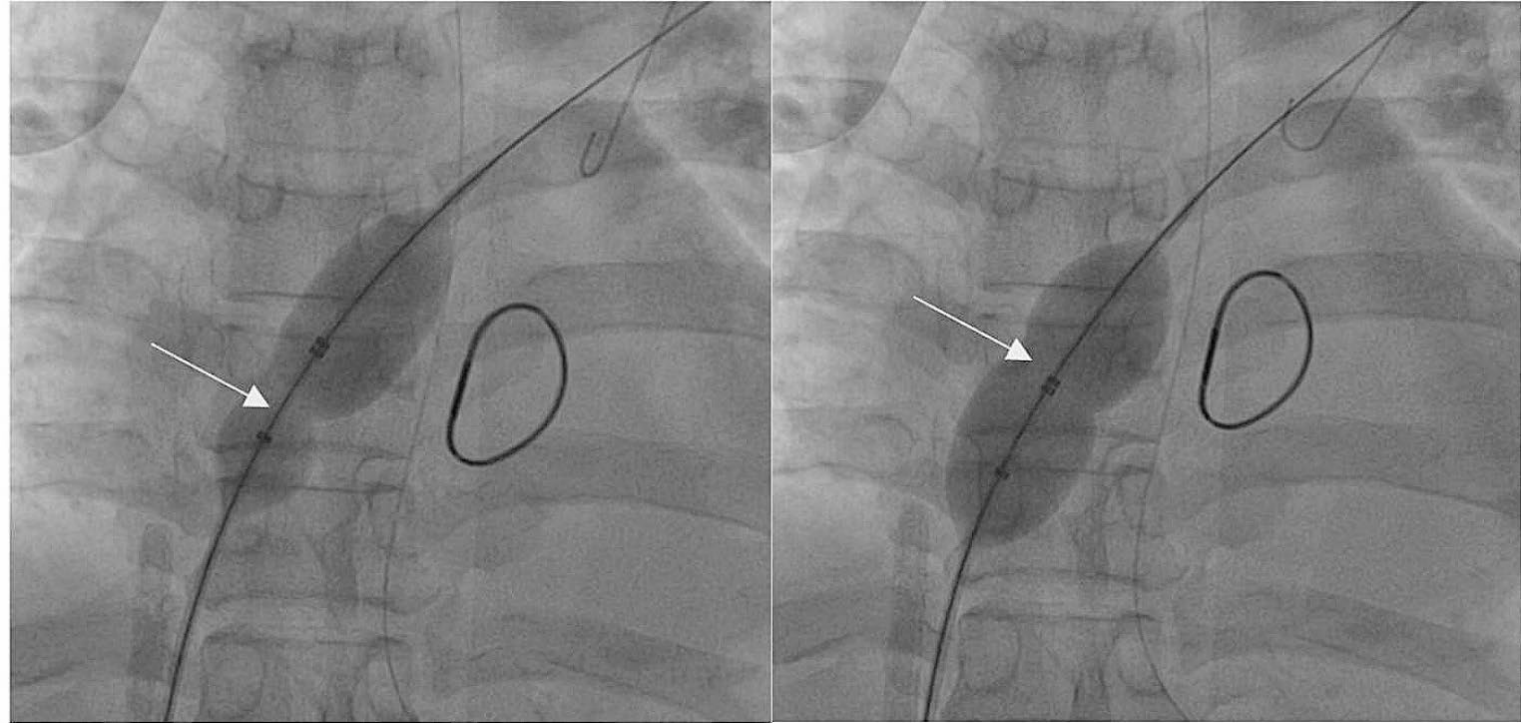
Percutaneous puncture balloon atrial septostomy. The white arrow indicates the “waist sign” of the balloon, indicating the position of the atrial septum

**Fig. 4:**
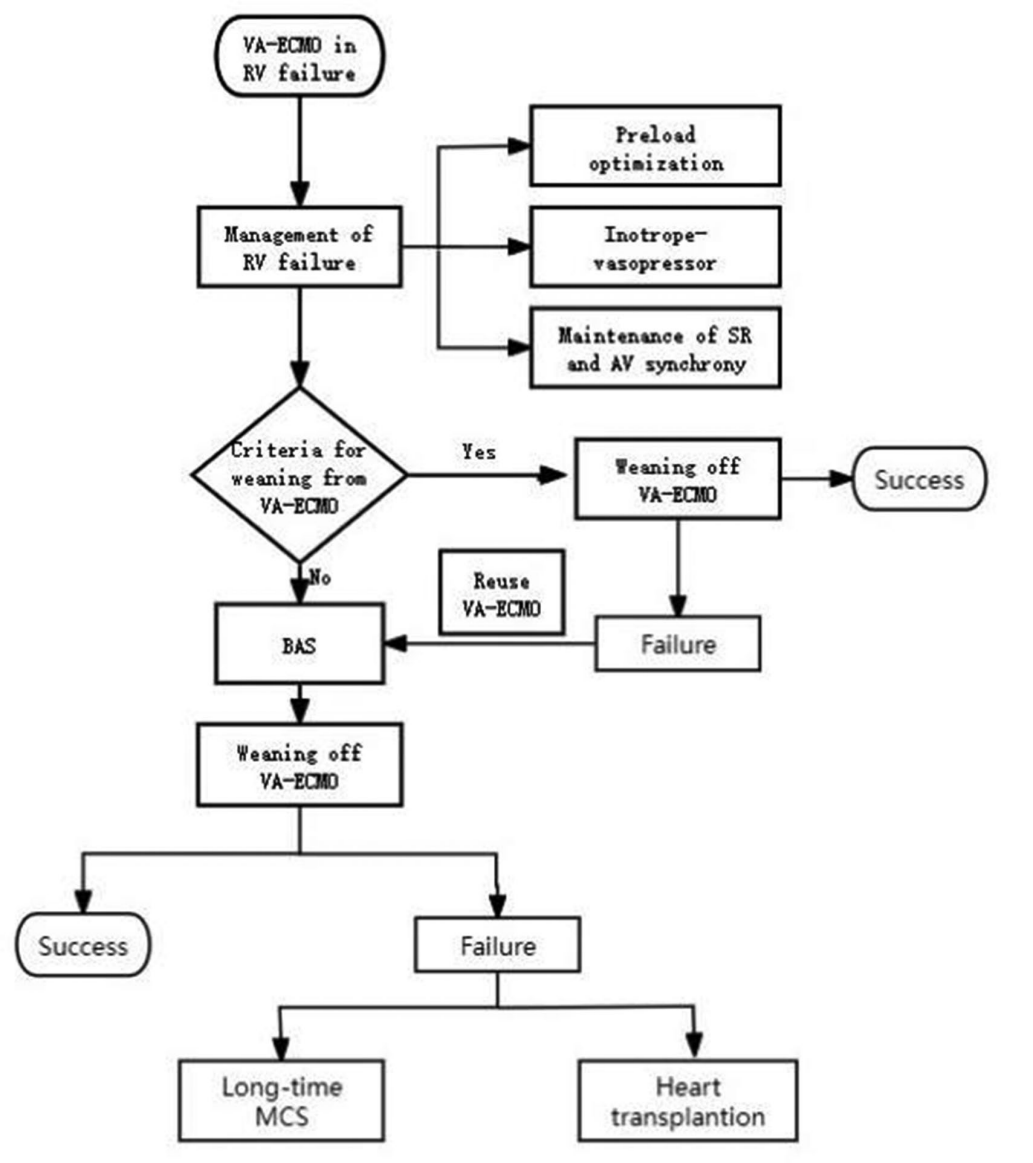
The management of right ventricular failure in patients undergoing extracorporeal membrane oxygenation. VA-ECMO: veno-arterial Extracorporeal Membrane Oxygenation; RV: right ventricular; SR: sinus rhythm; AV: atrioventricular; BAS: balloon atrial septostomy; MCS: mechanical circulatory support. Criteria for weaning from VA-ECMO [19]

To commence the procedure, it is necessary to puncture the femoral vein sheath on the opposite side of the VA-ECMO catheter, ensuring that the guide wire passes through the atrial septum. Subsequently, position the distal end of the guide wire at the termination point of either the upper or lower left pulmonary vein in order to establish a viable pathway.Adopt the left anterior oblique orientation to visualize the tangent position of the atrial septum, subsequently conducting selective left atrial angiography to visualize the atrial septum’s location and the openings of the left upper and/or left lower pulmonary veins. Subsequently, the balloon was inserted along the guide wire track, and the midpoint of the balloon was located in the atrial septum. A non compliant high-pressure vascular balloon (AMPLATZER Sizing Balloon II produced by Boston AGA Medical Corporation) with a diameter of 22 mm and a length of 33 mm was used. After the balloon was dilated and withdrawn, the right to left shunt beam width at the atrial septum ostomy site and the ostomy site was explored using echocardiography (Figs. 3, 4).

## Results

Among the four adult patients reported in this study who underwent Balloon atrial septostomy, one individual succumbed while the remaining three individuals survived. The findings from these three cases indicate that a brief duration of mechanical support may enable a significant proportion of patients with post-cardiotomy right ventricular dysfunction to achieve a successful bridge to recovery. In many instances, the right ventricle will exhibit a degree of recovery sufficient to generate adequate cardiac output during right-left shunting, even in the presence of significant right ventricle dysfunction. Three patients were observed in this study. The first patient had the longest survival time of four years. The second patient exhibited poor right heart function, congestion in the right heart system, moderate regurgitation of the mitral valve, severe regurgitation of the tricuspid valve, compromised right heart systolic function, and a low quality of life. The medical team is currently deliberating on the appropriate treatment for this patient. The third patient survived for one year and expressed satisfaction with herrecovery. They presented with a left-to-right shunt in the atrial septum, while their right heart function was predominantly normal. The fourth patient exhibited stenosis subsequent to undergoing mechanical mitral valve replacement surgery, characterized by a long disease duration, the presence of pulmonary hypertension, and significant impairment of right heart function. The condition experienced a relapse, prompting the patient’s family to discontinue treatment as a result of septic shock.

## Discussion

Right ventricular failure is a grave complication that arises from cardiac surgery, and it is linked to substantial morbidity and mortality. Devices for isolated RV support include micro-axial flow pumps, right ventricular assist devices(extracorporeal centrifugal flow or surgically implanted) and VA-ECMO. At present, there is only VA-ECMO in China. VA-ECMO, as a short-term mechanical circulatory aid, has a significant effect in the treatment of right heart failure, protecting the main organ functions of patients, especially liver and kidney function [11]. Following a period of typically 5–10 days for the patient’s myocardial function to recover, the withdrawal of VA-ECMO is undertaken. Some patients demonstrate dissatisfaction with the level of myocardial function restoration, as indicated by increased right atrial pressure, absence of urine production, and declining liver function following the cessation of VA-ECMO. However, the long-term effectiveness of mechanical circulatory support in the right heart remains insufficiently explored [12]. Additionally, it is an undeniable fact that the supply of donor hearts for transplantation is constrained. Some studies have reported on the utilization of half Fontan or bidirectional Green’s treatment as potential interventions for right heart failure [13, 14].

Balloon atrial septostomy (BAS) is an early developed interventional treatment technique. In 1966, Rashkind et al. [15] were the first to employ this technique for the palliative treatment of complete transposition of the great arteries, aiming to alleviate hypoxia and extend the lifespan of affected patients. Subsequently, the utilization of BAS gained prominence in the management of various complex congenital heart conditions marked by cyanosis, resulting in favorable outcomes in terms of palliative intervention. In 1983, Rich and Lam [16] made a significant contribution by reporting the initial use of transcatheter BAS as a palliative measure for individuals suffering refractory primary pulmonary hypertension and right heart failure. BAS has emerged as a feasible intervention for individuals diagnosed with severe pulmonary arterial hypertension who have exhibited insufficient response to pharmacological treatment or who are in the process of awaiting lung transplantation. The application of BAS in the context of VA-ECMO treatment elicited substantial scholarly attention throughout the 1990s. [26] In cases of extreme left ventricular failure during VA-ECMO support, left ventricular tension escalates and blood circulation becomes stagnant, impeding the restoration of left heart functionality. However, following the implementation of balloon atrial septal ostomy, blood is redirected from the left heart to the right heart, thereby facilitating left heart decompression[17, 18] The department has encountered multiple occurrences of utilizing BAS for VA-ECMO left heart decompression, leading to the conclusion that the technology has achieved a level of maturity. In an alternative scenario, the occurrence of right heart failure leads to an elevation in pressure within the right heart system, causing inadequate filling of the left heart system and compression of the left heart by a fully engorged right heart. Consequently, there is a notable reduction in cardiac output and challenges in the evacuation of VA-ECMO. Following the opening of the atrial septum during BAS, a right-to-left shunt develops, leading to a reduction in oxygen saturation but an increase in cardiac output, which may aid in the successful weaning of VA-ECMO. Among the 4 cases with severe right heart failure, 3 withdrew from ECMO and survived to this day. The first patient presented with ischemia affecting the right coronary artery and resulting in ischemic necrosis of the right ventricular myocardial cells, ultimately leading to the development of acute right heart failure. Despite the implementation of emergency surgical bypass, the right heart function was significantly impaired post-surgery, necessitating the utilization of circulatory assistance support. ECMO was withdrawn after the atrial septal stoma, and gradual improvement in the right ventricular function was observed. BAS exhibited bidirectional or left to right shunting. The second case was attributed to right heart failure resulting from an extended period of cardiopulmonary bypass and insufficient myocardial protection. Following BAS, VA-ECMO was discontinued. Presently, the right heart function is compromised, evident by pronounced congestion within the right heart system, and substantial regurgitation in the tricuspid valve, consequently leading to a diminished quality of life.The third instance of severe tricuspid regurgitation accompanied by right heart failure led to hemodynamic instability following the discontinuation of VA-ECMO. In order to alleviate the strain on the right heart, BAS was conducted, resulting in a gradual stabilization of circulation and obviating the need for further ECMO utilization. This patient’s right ventricular function has recovered well in the later stage, and we can communicate with the patient whether to undergo atrial septal defect occlusion surgery.We reflect on the fact that if the fourth patient underwent tricuspid valve surgery during remission, the prognosis may vary. There is controversy in cardiac surgery about whether surgery can improve the prognosis. The patient’s right heart systolic function decreased and the right heart load increased due to tricuspid valve regurgitation formed a vicious cycle. Nevertheless, the patient ultimately succumbed to septic shock.We have compiled the following treatment plans based on experience.

### Electronic supplementary material

Below is the link to the electronic supplementary material.

Supplementary Material 1

## Data Availability

No datasets were generated or analysed during the current study.
